# Killer-cell immunoglobulin-like receptors associate with HIV-1 infection in a narrow-source Han Chinese cohort

**DOI:** 10.1371/journal.pone.0195452

**Published:** 2018-04-17

**Authors:** Linghang Wang, Yonghong Zhang, Keyi Xu, Tao Dong, Sarah Rowland-Jones, Louis-Marie Yindom

**Affiliations:** 1 Beijing Ditan Hospital, Capital Medical University, Beijing, People's Republic of China; 2 Nuffield Department of Medicine, Headington, Oxford, United Kingdom; 3 MRC Human Immunology Unit, Weatherall Institute of Molecular Medicine, John Radcliffe Hospital, Oxford, United Kingdom; 4 Beijing You An Hospital, Capital Medical University, Beijing, People's Republic of China; University of Liverpool Institute of Infection and Global Health, UNITED KINGDOM

## Abstract

**Background:**

The HIV pandemic remains the most serious challenge to public health worldwide. The hallmark characteristics of the disease is the eventual failure of the immune system to control opportunistic infections and death. However not everyone who has HIV develops the disease at the same rate and so we are studying how the immune system works to control the virus in those who have been infected for decades and remain relatively healthy without the need of anti-retroviral therapy (ART).

**Methods:**

Genomic DNA samples from 513 Chinese Han individuals from Henan province were typed for 15 KIR and 3 HLA class I genes. Genotype frequencies were compared between a village cohort of 261 former plasma donors (SM cohort) infected with HIV-1 through an illegal plasma donor scheme who survived more than 10 years of infection without ART and 252 ethnically-matched healthy controls from a nearby village. KIR and HLA were molecularly typed using a combination of polymerase chain reaction (PCR) with sequence-specific primers (PCR-SSP) and sequence based techniques.

**Results:**

All 15 KIR genes were observed in the study population at various frequencies. *KIR2DL3* was significantly less common in the HIV-1 infected group (95.8% vs 99.2%, p = 0.021). The combination of *KIR3DS1* with homozygosity for HLA-Bw4 alleles (the putative ligand for *KIR3DS1*) was significantly less frequent in the HIV-1 infected group than in the control group (6.0% vs 12.0% respectively, p = 0.023).

**Conclusion:**

Specific KIR-HLA compound genotypes associate with differential outcomes to infection and disease progression following exposure to a narrow-source HIV-1.

## Introduction

Natural Killer (NK) cells play a bridging role between innate immunity and adaptive immunity to control viral infection and malignant diseases. NK cells can be rapidly activated in the absence of previous antigen sensitisation and therefore represent a crucial first line of defence against viral infections [1]. Whether or not a NK cell is activated is determined by the balance of an array of activating signals and inhibitory signals transduced from their cell-surface receptors. Of all the NK cell receptors, KIRs are by far the most polymorphic and could therefore explain differential responses to viral infections between individuals.

The KIR gene family is encoded within a 100–200 Kb region of the Leukocyte Receptor Complex (LRC) located on human chromosome 19, and consists of 15 functional gene loci and 2 pseudogenes [2]. The extensive diversity within the *KIR* system is found at several levels: locus, allele, ligands and within their expression patterns. The expression of KIRs on NK cells is stochastic and variegated [3,4]. Potentially all these levels of KIR variation can influence the host immune responses to infection.

KIR molecules are classified according to the number of extra-cellular domains, as either KIR2D or KIR3D molecules. KIR molecules are also divided into L or S, representing long or short cytoplasmic tails, respectively. Broadly speaking, KIR2DL and KIR3DL genes encode inhibitory molecules (with the exception of *KIR2DL4* which has both inhibitory and activation potential), whereas KIR2DS and KIR3DS encode activating receptors. At the haplotype level, KIR genes segregate in a unique manner into two distinctive groups namely A and B that are maintained in different world populations by balancing selection [5,6]. Haplotype A is relatively simple with nine genes encoding predominantly inhibitory receptors, with only one activating gene (*KIR2DS4)* if expressed. Conversely the B haplotypes constitute a more extensive grouping that contain a mixture of activating and inhibitory KIRs. Present in each haplotype are four framework genes (*KIR3DL3*, *KIR3DP1*, *KIR2DL4* and *KIR3DL2)* that delimit three framework regions namely: the centromeric part from *KIR3DL3* to *KIR3DP1*, the central region from *KIR3DP1* to *KIR2DL4*, and the telomeric part from *KIR2DL4* to *KIR3DL2* [7,8]. The central part is the recombination hotspot while both the centromeric and telomeric regions have variable gene-content.

KIR molecules regulate the activity of NK and some T cells through interaction with specific Human Leucocyte Antigen (HLA) class I molecules which are their principal ligands. A functional interaction can only occur when both ligands and receptors are co-expressed in the same individual.

Several reports have described associations between particular KIR-HLA combinations and clinical outcome in HIV infections [9–15]. A compound genotype comprising KIR3DS1 and Bw4-80I (isoleucine at position 80 of the corresponding HLA-B alleles) was shown to have a protective effect against AIDS progression in ART naive HIV-1 infected individuals [13]. This compound genotype was further shown to correlate with lower viral load and protection from opportunistic infections [14]. However the protective effect of this compound genotype was not seen in two other studies [11,16]. Recent evidences suggest that KIR3DS1 binds to HLA-F [17,18] but no direct binding to *HLA-B Bw4-80I* has so far been demonstrated [19,20]. Nevertheless, a functional study showed NK cells expressing KIR3DS1 more potently inhibited HIV replication in target cells expressing HLA-B Bw4-80I compared with KIR3DS1 negative cells [21]. Surprisingly, in the first cohort, the combination of KIR3DL1*h/y + HLA-B Bw4-80I was also shown to be protective against HIV progression [22]. All of these studies suggest that NK cells expressing KIR molecules that interact with their HLA ligands might play an important role in the outcome of HIV-1 infection.

In this study we have examined the relationship between KIR polymorphism and clinical outcome in a unique village cohort of HIV-1-infected former plasma donors (FPDs) in Henan province, China. The cohort was established in a single village in 2004, when most of the individuals had been living with HIV-1 for more than 10 years without anti-retroviral therapy (ART), and could therefore be regarded as “slow progressors”. This cohort provides particular advantages for genetic association studies because most of the major factors that are known to affect the natural history of HIV-1 infection, such as viral strain, transmission route and timing of infection, were very similar amongst all participants, as previously described [23]. Although no samples were available from rapid progressors who died from HIV-1 complications prior to 2004, we compared HLA and KIR frequencies in the HIV-1 cohort with those in a nearby village which did not participate in the plasma donation scheme. Our hypothesis was that if specific KIR genes or KIR-HLA combinations provide protection against HIV-1 progression, they may have become enriched in these “slow progressors” compared to the general population.

## Materials and methods

### Study populations

SM village is an isolated rural community in Henan province in central China. Between 1994 and 1995, many villagers in this community participated in a paid plasma donation scheme which became contaminated with HIV-1, thought to be due either to contamination of blood collection equipment or pooled red cells being returned to donors [24]. The SM cohort was established in 2004, when most of the HIV-infected former plasma donors (FPD) had survived with HIV for more than 10 years in the absence of ART. 149 premature deaths had been recorded in the village with symptoms compatible with HIV-1 disease before 2004. To a certain extent all of the 261 individuals in the SM cohort can be regarded as “slow progressors” [25–27]. Phylogenetic analysis of the infecting virus, studying the p17 region of *gag* and C2-V3 regions of *env*, suggested that the paid plasma donation associated HIV-1 subtype B′ epidemic in central China is monophyletic [28]. Based on the epidemiological history and further phylogenetic analysis of the HIV-1 *Gag*, *Pol*, and *Nef* pro-viral sequences in the SM FPDs, the SM village outbreak was also considered to be a narrow-source infection [23]. In order to compensate for the loss of “rapid progressors” from the cohort, 252 healthy Han Chinese donors were recruited from a nearby village that had not taken part in the illegal plasma donation scheme to provide population HLA and KIR frequencies.

This study was approved by the Ethics committees of Beijing You’an hospital and Oxford University (OxTREC). All the participants provided their written informed consent with the assistance of medical staff prior to sample collection.

### DNA isolation

Genomic DNA was extracted from 300ul of whole blood containing EDTA, using a DNA isolation kit according to the manufacturer’s instructions, and quantified by Nanodrop spectrometry.

### KIR and HLA class I genotyping

Genomic DNA was genotyped using a polymerase chain reaction- sequence specific priming (PCR-SSP) technique to detect the presence or absence of the following inhibitory genes: *KIR2DL1*, *KIR2DL2*, *KIR2DL3*, *KIR2DL4*, *KIR2DL5*, *KIR3DL1*, *KIR3DL2*, *KIR3DL3;* activating genes: *KIR2DS1*, *KIR2DS2*, *KIR2DS3*, *KIR2DS4*, *KIR2DS5*, *KIR3DS1*, and pseudogene *KIR2DP1* as previously described [29]. HLA class I genotyping was performed using sequence-based techniques. Briefly, locus-specific primers flanking exons 2 and 3 were used to amplify each locus and two other pairs of internal primers were used to sequence the purified PCR products in both directions with a BigDye Terminator version 3.1 cycle sequencing kit (Applied Biosystems, Foster city, CA).

### Statistical analysis

Stata version 14.1 (StataCorp, Texas, USA) was used to perform all statistical analyses. Frequencies of genes, alleles, genotypes and haplotypes were determined using direct counting. Heterogeneity in frequencies of genes, alleles, genotypes and haplotypes between HIV-infected and HIV-uninfected groups was determined using Chi-squared or Fisher’s exact tests as may be appropriate. P-values of <0.05 after Bonferroni correction for multiple testing were considered significant.

## Results

### Demographic and clinical characteristics of participants

Blood samples were collected from all 261 identified former plasma donors in the SM village with chronic HIV-1 infection. Demographic and clinical characteristics are available for 258 treatment naïve adults with mean age of 43 years, 56% of whom were females (Table 1).

**Table 1:** Characteristics of surviving HIV-1 infected individuals at enrolment.

Gender	Total number (n = 258)	Mean±SD (%)
Male	113	43.80%
Female	145	56.20%
Age (years)	258	42.98 ± 9.84 (27–60)
Duration of Infection (years)	258	14.56 ± 1.42 (12–16)
Route of infection / therapy	Total number (n = 258)	%
Plasma donation	258	100%
Other	0	0
ART before 2004	0	100%
ART after 2004	121	46.90%
Viral load* / CD4 counts†	Total number	%
VL < 50	27	29.03%
VL ≤ 2000 (≤3.3log)	8	8.60%
VL > 2000 (>3.3log)	58	62.37%
CD4 ≥ 500	81	34.03%
200 ≥ CD4 < 500	87	36.56%
CD4 < 200	70	29.41%

*: Viral loads were measured and reported as copies per mL of blood collected from 93 participants

†: CD4+ T cell counts were performed and reported as number of CD4^+^ T cells per mL of blood, 238 participants had baseline CD4 data.

Two hundred and thirty-eight HIV-1 infected participants had baseline CD4 count data and 93 had viral load measured at recruitment with 29% being undetectable. The mean duration of infection without any form of anti-retroviral therapy was 14.6 years with a substantial number (34%) still relatively heathy with CD4 counts above 500 cells/mL.

### KIR gene frequencies in the Han Chinese cohorts

We report the frequencies of 15 KIR genes in a unique cohort of HIV-1 infected and uninfected Henan Han Chinese population. We observed that all three frame work genes investigated namely *KIR2DL4*, *KIR3DL2* and *KIR3DL3*, were present in all 252 control (uninfected) participants and in nearly all (>99.2%) HIV-1 infected individuals. Other genes present at high frequencies (>97%) in the uninfected group included two inhibitory genes (*KIR2DL1* and *KIR2DL3)*, one activating gene (*KIR2DS4)*, and the pseudogene (*KIR2DP1)*. In addition to the aforementioned genes, *KIR3DL1* frequency was high in the HIV-1 infected group (Table 2).

**Table 2:** KIR frequency distribution between SM patients and healthy controls.

KIR gene	SM patients %	Healthy control %	P
	n = 261	n = 252	
*KIR2DL1*	98.5	100	0.124 F
*KIR2DL2*	22.6	21.8	0.915
*KIR2DL3*	95.8	99.2	0.021^*^
*KIR2DL4*	99.2	100	0.499 F
*KIR2DL5*	39.5	42.9	0.435
*KIR2DS1*	34.9	37.3	0.566
*KIR2DS2*	21.5	20.6	0.820
*KIR2DS3*	13.8	17.9	0.207
*KIR2DS4*	98.1	97.2	0.571 F
*KIR2DS5*	28.7	29.4	0.875
*KIR3DL1*	97.3	94.1	0.068
*KIR3DL2*	100	100	-
*KIR3DL3*	100	100	-
*KIR3DS1*	31	38.1	0.096
*KIR2DP1*	98.5	100	0.124 F

Pearson Chi-Square was used to perform the comparison.

*: Fisher’s Exact Test. P<0.05 was significant.

–represents no comparison.

In general most activating KIRs (except KIR2DS4) were significantly less frequent compared to their inhibitory counterparts with the least frequent being *KIR2DS3* (13.8% and 17.9% in cases and controls, respectively).

### KIR2DL3 associates with non-protection against rapid disease progression

Comparative analysis of individual KIR genes between cases and controls revealed that the inhibitory gene *KIR2DL3* was significantly overrepresented in the uninfected group (99.2% vs. 95.8% in control and cases, respectively, corrected P-values = 0.021). Given that the HIV+ group (n = 261) is enriched with long-term non-progressors and slow progressors, this finding may suggests that a disproportionate number of *KIR2DL3+* individuals who participated in the blood donation scheme in the SM village may have died prior to 2004 when this cohort was established. It may be worth mentioning here that *KIR2DL3* is part of a dimorphic group (KIR2DL2/L3) with both genes occupying the same locus on human chromosome 19. We did not see this effect with *KIR2DL2* which was equally distributed between groups (S1 Table). The underrepresentation of *KIR2DL3* among HIV-1 survivors (slow progressors) and its plausible association with HIV disease progression is independent of its dimorphic nature.

### KIR gene frequencies are similar between Henan Chinese Han and other Han populations

Here, we compared KIR frequencies in the uninfected Henan Chinese Han that participated in the present study (n = 252) to those of other Chinese Han populations including Zhejiang Chinese Han [30] and Jiangshu Chinese Han [31]. Our observed KIR frequencies were also compared against those reported by Miyashita and colleagues in a Japanese population [32] (S1 Fig). With the exception of *KIR2DL5* and *KIR2DS4*, only marginal differences were observed between the three Chinese Han populations and Japanese. The frequency of *KIR2DL5* is highly variable between populations: lower in Zhejiang Chinese Han (30.8%) and higher in the Japanese (48.5%). This difference, however, disappeared after correction for multiplicity testing by the Bonferroni’s method. A highly significant difference was seen with *KIR2DS4*, which was significantly less frequent in the Japanese population (87%) compared to Chinese Han (S1 Fig). There was also some variability in the frequency of KIR2DS4 amongst Chinese Han populations. Whereas every Chinese Han in Zhejiang has KIR2DS4, 7.3% of Jiangshu Chinese Han individuals lack this activating gene.

### KIR genotype/haplotype distribution and association with disease

Using the number of KIR genes present in each participant, we computed individual KIR genotypes and compared with existing data in public databases such as the “Allele*frequency.net” [33]. In the present study, 54 genotypes were identified in 513 individuals. S1 Table shows the frequencies of most frequent genotypes (present in at least 1% of the study population). Genotype 1 is the most common genotype worldwide and was present in 50% of our samples.

### HLA class I gene frequencies in HIV-1-infected and uninfected groups

We generated HLA class I data on 233 cases and 252 controls. Table 3 shows the distribution of HLA class I genotypes detected in our study population.

**Table 3:** HLA class I frequencies between cases and controls.

HLA class I	SM patients(n = 233) %	Healthy controls(n = 252) %	P
HLA-A alleles			
A*01	10.3	6.8	0.164
A*02	53.2	47.8	0.235
A*03	8.2	6.8	0.563
A*11	24.5	26.3	0.644
A*24	24.5	29.9	0.182
A*26	2.6	6.4	**0.045**
A*31	5.6	6.4	0.713
A*32	6.4	4.8	0.428
A*33	21.0	24.7	0.338
HLA-B alleles			
B*07	7.7	9.2	0.571
B*08	2.6	2.0	0.668
B*13	21.0	26.3	0.174
B*15	17.6	20.3	0.446
B*27	7.3	4.0	0.113
B*35	6.9	5.6	0.557
B*37	3.9	2.8	0.510
B*38	5.6	6.8	0.587
B*39	3.9	4.0	0.945
B*40	29.2	25.5	0.363
B*44	13.7	17.5	0.252
B*46	9.4	10.0	0.848
B*48	5.6	4.4	0.545
B*51	14.2	16.7	0.436
B*52	8.2	7.2	0.685
B*54	6.4	5.2	0.554
B*55	2.2	3.6	0.346
B*57	4.7	3.2	0.386
B*58	11.6	11.6	0.991
HLA-C alleles			
Cw*01	18.6	18.3	0.942
Cw*02	5.8	2.0	**0.032**
Cw*03	33.6	39	0.22
Cw*04	9.7	10.8	0.714
Cw*06	29.7	27.9	0.672
Cw*07	28.8	29.1	0.938
Cw*08	22.6	16.3	0.085
Cw*12	11.1	9.2	0.492
Cw*14	10.6	13.9	0.271
Cw*15	11.1	13.9	0.344

At the group specific levels (2-digit HLA data), we found 9 HLA-A (01, 02, 03, 11, 24, 26, 31, 32, and 33) with HLA-A*02 being the most common in both groups. A*26 tended to be slightly overrepresented in the uninfected control group compared to cases (6.4% vs. 2.6%, respectively). Similarly, 19 different HLA-B groups of alleles were present in our study population, albeit with a similar distribution between cases and controls, and HLA-B*40 and HLA-B*13 were the most common. Among the 10 HLA-C group of alleles that were detected in this study, HLA-Cw*03 was the most frequent (39.0% and 33.6% in controls and cases respectively). However, HLA-Cw*02 was marginally overrepresented in cases compared to controls (Table 3).

### Centromeric and telomeric KIR motifs and genotypes in HIV-1-infected and uninfected groups

We adopted the recent techniques and terminologies used by Cooley *et al* [34] and Pyo *et al* [35] to assign centromeric (cen) and telomeric (tel) motifs and genotypes to each study sample. Comparisons of the group of genes present at the telomeric and centromeric parts of the KIR gene loci were performed between the two groups. The frequencies of most telomeric and centromeric motifs as well as genotypes were very similar between the SM patient group and the healthy control group (Table 4). However, a significant difference was observed amongst people carrying the telomeric genotype *B1/Bx* (with Bx representing other *Tel-B* genotypes), which was significantly less frequent in the SM patient group (0.4%) compared to the healthy control group (2.8%).

**Table 4:** Centromeric and telomeric motif and genotype distribution in the study population.

	SM patients (%)	Healthy controls (%)	P
Centromeric motifs			
cen-A	72.8	75.8	0.438
cen-B1	6.5	3.6	0.129
cen-B2	14.2	16.7	0.435
cen-B3	23	23.4	0.909
cen-Bx	6.1	4.4	0.371
Telomeric motifs			
tel-A	96.9	94.1	0.115
tel-B1	25.3	32.9	0.570
tel-Bx	16.1	12.7	0.275
Centromeric genotype			
c-A/A	52.5	52.4	0.980
c-A/B1	4.6	3.6	0.558
c-A/B2	13	16.7	0.246
c-A/Bx	2.7	3.2	0.741
c-B1/B1	0.4	0	1.000
c-B1/Bx	1.5	0	0.124
c-B2/B2	1.2	0	0.249
c-B3/B3	22.2	23	0.830
c-B3/Bx	0.8	0.4	1.000
c-Bx/Bx	1.2	0.8	1.000
Telomeric genotype			
t-A/A	59	57.1	0.670
t-A/B1	23	27.4	0.252
t-A/Bx	14.9	9.5	0.062
t-B1/B1	1.9	2.8	0.519
t-B1/Bx	0.4	2.8	**0.035**
t-Bx/Bx	0.8	0.4	1.000

Cen: centromeric; Tel: telomeric; *Cen-Bx*: other *Cen-B* genotypes except *Cen-B1*, *Cen-B2* and *Cen-B3*. *Tel-Bx*: other *Tel-B* genotypes except *Tel-B1*.

### Compound KIR-HLA genotype comparison between HIV-1-infected and uninfected groups

Using known KIR-HLA ligand-receptor relationships, we computed compound genotypes for each study participant and compared their frequencies between groups. We found that the compound genotype *KIR3DS1* + *HLA-Bw4* was relatively less frequent in the SM patient group (6%) than in the healthy control group (12%) (Table 5).

**Table 5:** KIR-HLA compound genotype distribution.

KIR-HLA*	SM patients group (%)	Healthy control group (%)	P
3DS1+Bw4/x	25.3	29.9	0.263
3DL1+Bw4/x	73.0	71.3	0.687
3DL1S1+Bw4/x	74.3	75.7	0.713
3DS1+Bw4/Bw4	6.0	12.0	**0.023**
3DL1+Bw4/Bw4	22.3	22.0	0.831
3DL1S1+Bw4/Bw4	22.3	23.9	0.680
2DL2+C1/x	20.4	19.9	0.906
2DL3+C1/x	86.7	90.0	0.258
2DL2/3+C1/x	90.3	90.0	0.934
2DS2+C1/x	19.5	18.7	0.837
2DL2+C1/C1	10.2	10.0	0.937
2DL3+C1/C1	46.5	48.6	0.640
2DS2+C1/C1	9.3	9.6	0.920
2DL1+C2/x	50.0	51.0	0.828
2DS1+C2/x	22.6	19.1	0.355

*: x represents Bw4, Bw6, C1, or C2

## Discussion

HIV-1 infection represents a complex interaction between a polymorphic virus and a polymorphic host immune system over many years of infection. In the attempt to understand why HIV-1 outcomes can differ so markedly between individuals, immunogenetic studies provide an *in vivo* population view of factors that affect the host response to the virus, providing suggestions for subsequent *in vitro* functional studies.

There have been few previous studies of the immunogenetic associations of HIV-1 infection in Chinese people. The SM cohort presents several unique advantages for genetic studies: firstly, almost all the individuals in the SM patient group were infected during a very short period of time, between 1993 and 1995 when the paid plasma donation scheme was prevalent in rural areas of Henan province; secondly, based on the analysis of the infecting HIV-1 sequences [23], the cohort as a whole was infected by a relatively narrow source virus and occurred only in individuals with an evident plasma donation history, which strongly suggests that they were infected via the same blood contamination route. Hence, one side of the virus-host dynamic is relatively constant, which provides a unique advantage to evaluate how the other side, host factors, will shape the outcome of HIV-1 infection. Previous studies in the same cohort showed that CTL pressure has a major effect on inter-host HIV-1 viral diversity and is therefore thought to represent a key element of viral control [23]; however, CTL pressure alone accounted for only around 50% of the observed viral variant, implicating the involvement of additional components of the immune system in HIV-1 control. Moreover, HLA class I molecules, as the central part of the CTL immune response, are also the major KIR ligands, which highlights the potential role for KIRs in defence against HIV-1 infection.

One of the unavoidable problems encountered in this analysis, which is often seen in this kind of retrospective study, is that the ‘rapid progressors’ at one end of the progression spectrum have already died and are, therefore, missing due to frailty bias, which may limit the power of comparison. Although we acknowledge the limitations, recruitment of healthy controls from a geographically adjacent village have to some extent compensated for this lack of power. On the other hand, it is appreciated that this healthy control group was not recruited with the specific aim of studying susceptibility to HIV-1 infection.

Our first analysis described the profile of KIR gene distribution in the two groups of Chinese Han. No significant differences were seen for most of the KIR genes between the groups, except for *KIR2DL3*. *KIR2DL3* was previously shown to be associated with an increased likelihood of viral clearance following acute hepatitis C viral infection [36]. The presumed mechanism underlying this observation is that the weaker inhibitory interaction of *KIR2DL3* with *C1*, but not the stronger inhibitory interaction of *KIR2DL2* and *KIR2DL1* with *C1* and *C2*, could allow penetrance of activating signals during viral infection. In our study, a significant difference of *KIR2DL3* frequency was also detected between the “slow progressors” and the healthy controls. Since it is necessary to incorporate the KIR ligand interactions into a biological model rather than considering KIRs in isolation, we further investigated *KIR2DL3*+ *C1C1* or *C1C2* between the two groups, but no further significant differences were found. Hence the initial difference has to be considered with caution. In the healthy controls, the frequency of *KIR2DL3* was 99.2%, which was similar to that reported in other two Chinese Han populations, at 99% and 100%, respectively [30,31]: however, in the SM patient group the frequency of *KIR2DL3* (95.8%) was significantly lower. The KIR gene frequencies were generated using SSP-PCR typing, which relies on sequence-specific primers to amplify specific KIR genes. It is plausible that variation in the binding positions of these “sequence-specific” primers could lead to a reported absence of the gene if rare alleles of KIR2DL3 were present, although it would be surprising for this only to occur in the HIV-1-infected cohort.

The predominant variation between KIR genotypes is in the number of genes, leading to distinct A and B haplotypes [37]. The A haplotype can be identified by the presence of *KIR2DS4* as the only activating receptor gene, whereas B haplotypes express multiple activating receptors, but not *KIR2DS4* [38]. In general, A haplotypes have been associated with an improved response to pathogens, whereas B haplotypes are linked with improved reproductive fitness [39–41]. In our study, the A haplotype (genotype 1 in S1 Table) accounted for 50% of SM patients, at a similar frequency to that (51%) in healthy controls.

The *KIR3DL1/S1* gene is distinct due to its diversity and varied expression patterns. It is the only KIR locus that encodes both inhibitory and activating allotypes. KIR3DL1 recognises HLA-B and HLA-A alleles that express the Bw4 serological motif. Although *KIR3DL1* and *KIR3DS1* share high similarity and Bw4 has been proposed to be the ligand for *KIR3DS1*, no direct binding of *KIR3DS1* and Bw4 has yet been demonstrated. However, several epidemiological and functional studies support some kind of interaction between *KIR3DS1* and *HLA-B Bw4-80I* in the NK cell response to HIV infection. Martin and colleagues reported that *KIR3DS1*+*HLA B Bw4-80I* was protective against HIV progression [13], but this has not been found in other studies: indeed, Gaudieri *et al* reported that individuals with *KIR3DS1* and *Bw4-80I* actually exhibited an accelerated progression to AIDS [11] whilst O’Connell *et al* noted there was no correlation between KIR/HLA genotype and control of HIV replication by NK cells *in vitro* in a small cohort of elite controllers [42]. In our study, the frequency of *KIR3DS1*+ *Bw4* homozygotes was significantly lower in the SM “slow progressors” group (6%) compared to that (12%) in the healthy group. This observation implies that there has been a loss of this compound genotype in the “slow progressors”, which could either imply that this combination does not mediate delayed HIV progression, or alternatively, that the presence of this compound genotype protects against blood-borne HIV-1 infection. In our study, we were unable to assign each *Bw4* motif to 80I or 80T subgroups, so could not investigate the effect of *KIR3DS1*+*Bw4-80I* in this cohort.

*KIR3DL1* shows extensive polymorphism, and its variation has functional significance in terms of cell surface expression levels and inhibitory capacity, which was shown to be relevant in a previous association study with HIV infection [22]. However in our study there was no correlation with *KIR3DL1/Bw4* genotype and HIV progression.

Additional confounders of this study might include limitations in the sample sizes used for the two cohorts. Despite the relatively small sample sizes, the “genetic association effect” could potentially be magnified because other confounders such as ethnicity, infecting founder viruses, transmission routes and infection durations are largely controlled for in the SM cohort.

It is becoming apparent that host genetic variation can exert significant selective pressure on the virus [43]. The advantage of genetic epidemiological studies is that they can provide an *in vivo* population view of factors that affect the host response differentially across humans, giving meaning to subsequent functional studies to further investigate the molecular basis of their impact on AIDS pathogenesis and help clarify the relative contribution of the innate and adaptive immune response, which may lead in turn to the development of better therapies and vaccines.

## Supporting information

S1 Fig KIR gene frequencies in Henan Chinese Han and neighbouring populations. Zhejiang Chinese Han [30], Jiangshu Chinese Han [31], and Japanese [32]. **: P = 5.3x10^-6^
(DOCX)

S1 Table KIR genotype distribution in the SM cohort. *: Genotype identification numbers are unique numbers assigned as per available data in AlleleFrequency in worldwide population database.
(XLS)
