# Supplementary material for: Killer-cell immunoglobulin-like receptors associate with HIV-1 infection in a narrow-source Han Chinese cohort
Source: PLoS One. 2018 Apr 17;13(4):e0195452. doi: 10.1371/journal.pone.0195452 (PMC5903672; doi:10.1371/journal.pone.0195452)
Supplement: S1 Fig — Zhejiang Chinese Han [30], Jiangshu Chinese Han [31], and Japanese [32]. **: P = 5.3x10^-6^ (DOCX) [file pone.0195452.s001.docx]

S1 Fig: KIR gene frequencies in Henan Chinese Han and neighbouring populations


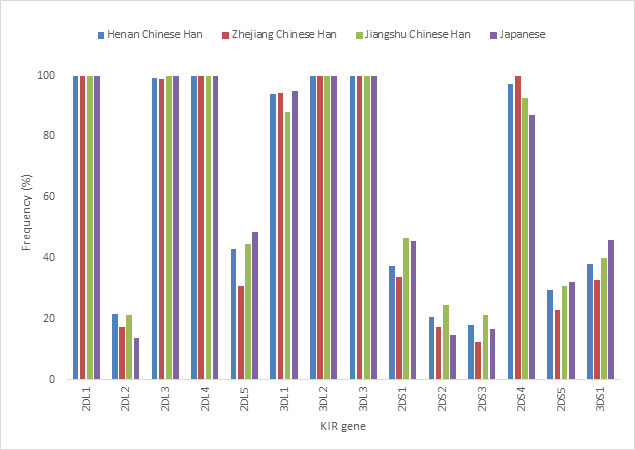


Zhejiang Chinese Han [[30](#_ENREF_30)], Jiangshu Chinese Han [[31](#_ENREF_31)], and Japanese [[32](#_ENREF_32)]. **: P = 5.3x10^-6^

S1 Table: KIR genotype distribution in the SM cohort

| Genotype ID* | 3D  L3 | 2D  S2 | 2D  L2 | 2D  L3 | 2D  L5 | 2D  S3/5 | 2D  P1 | 2D  L1 | 2D  L4 | 3D  L1 | 3D  S1 | 2D  S1 | 2D  S4 | 3D  L2 | Control  n (%) | Case  n (%) | OR (95% CI) | P |
| --- | --- | --- | --- | --- | --- | --- | --- | --- | --- | --- | --- | --- | --- | --- | --- | --- | --- | --- |
|  |  |  |  |  |  |  |  |  |  |  |  |  |  |  |  |  |  |  |
| 1 |  |  |  |  |  |  |  |  |  |  |  |  |  |  | 129 (51.2) | 132 (50.6) | 0.98 (069-1.38) | 0.889 |
| 28 |  |  |  |  |  |  |  |  |  |  |  |  |  |  | 46 (18.3) | 47 (18.0) | 0.98 (063-1.54) | 0.942 |
| 382 |  |  |  |  |  |  |  |  |  |  |  |  |  |  | 13 (5.2) | 19 (7.3) | 1.44 (0.70-2.99) | 0.321 |
| 6 |  |  |  |  |  |  |  |  |  |  |  |  |  |  | 19 (7.5) | 10 (3.8) | 0.49 (0.22-1.08) | 0.069 |
| 4 |  |  |  |  |  |  |  |  |  |  |  |  |  |  | 9 (3.6) | 10 (3.8) | 1.10 (0.43-2.70) | 0.876 |
| 55 |  |  |  |  |  |  |  |  |  |  |  |  |  |  | 5 (2.0) | 9 (3.4) | 1.76 (0.58-5.35) | 0.309 |
| 200 |  |  |  |  |  |  |  |  |  |  |  |  |  |  | 3 (1.2) | 5 (1.9) | 1.62 (0.38-6.87) | 0.508 |
| 14 |  |  |  |  |  |  |  |  |  |  |  |  |  |  | 3 (1.2) | 4 (1.5) | 1.29 (0.29-5.84) | 0.739 |
| 25 |  |  |  |  |  |  |  |  |  |  |  |  |  |  | 3 (1.2) | 3 (1.1) | 0.97 (0.19-4.83) | 0.966 |
| 70 |  |  |  |  |  |  |  |  |  |  |  |  |  |  | 3 (1.2) | 2 (0.8) | 0.64 (0.11-3.88) | 0.625 |
| 75 |  |  |  |  |  |  |  |  |  |  |  |  |  |  | 4 (1.6) | 1 (0.4) | 0.24 (0.03-2.16) | 0.166 |

*: Genotype identification numbers are unique numbers assigned as per available data in AlleleFrequency in worldwide population database.
